# Skin cancer risk perception and sun protection behavior at work, at leisure, and on sun holidays: a survey for Danish outdoor and indoor workers

**DOI:** 10.1186/s12199-018-0736-x

**Published:** 2018-10-02

**Authors:** Kasper Grandahl, Kristina Sophie Ibler, Gunnar Hellmund Laier, Ole Steen Mortensen

**Affiliations:** 1 The Department of Occupational Medicine, Copenhagen University Holbaek, Gl. Ringstedvej 4B, 4300 Holbaek, Denmark; 2 The Department of Dermatology, Zealand University Hospital, Roskilde, Denmark; 3 PFI (Production, Research, Innovation) Region Zealand, Sorø, Denmark; 4 Section of Social Medicine, Department of Public Health, University of Copenhagen, Copenhagen, Denmark

**Keywords:** Workplace, Risk behavior, Behavioral study, Sunscreen use, ICNIRP, UV, OSC, UV exposure, Ultraviolet radiation, Denmark, Preventive action

## Abstract

**Background:**

To prevent occupational skin cancer, it is essential that the sun-protective behavior of outdoor workers is adequate. The aim is to study the sun-protective behavior of Danish outdoor workers at work, at leisure, and on sun holiday and compare it to that of indoor workers.

**Methods:**

This is a cross-sectional study, based on a 53-item survey completed by Danish outdoor (*n* = 380) and indoor workers (*n* = 119) in 2016–2017. Status as outdoor or indoor worker was decided based on self-report and behavioral differences were tested using (paired) *t* tests and multiple regression adjusted for age, sex, educational level, history of smoking, and skin type.

**Results:**

Danish outdoor workers at work use sun protection less than they do at leisure and on sun holiday (*α* < .05) where their sun protection behavior is similar to that of indoor workers. The proportion of Danish outdoor workers that always/often use sun protection at work is for shade seeking around noon 4.2%, sunscreen 34.5%, wide-brimmed hat 25.3%, and long trousers and shirt with sleeves 42.4%. Of Danish outdoor workers, 49.5% do not think about the risk of occupational skin cancer and 11.8% think the risk is insignificant, 32.4% think that the use of sun protection is of low or no importance, 84.2% consider sunburn important as skin cancer risk factor still 88.9% have a history of sunburn at work, > 80.0% agree that risk of skin cancer is reduced by the use of sun protection, and only 4.0% dismiss the possibility of sun protection use at work.

**Conclusions:**

Skin cancer risk and use of sun protection at work are largely neglected in Danish outdoor workers, more so than at leisure and on sun holiday where their risk behavior resembles that of indoor workers. This indicates an untapped workplace preventive potential.

**Electronic supplementary material:**

The online version of this article (10.1186/s12199-018-0736-x) contains supplementary material, which is available to authorized users.

## Background

Solar ultraviolet (UV) radiation is the main cause of skin cancer, a major health problem throughout Europe [1]. The only way to rectify this problem is by better use of sun protection.

For outdoor workers, exposure is habitual during the hours around solar noon where solar UV radiation is most intense. International studies show high-level solar UV radiation exposure at work and increased risk of skin cancer in outdoor workers compared to indoor workers [1–6]. However, in 2010, the Danish Cancer Society published a registry based case-control study showing a low risk of skin cancer in Danish outdoor workers compared to wage-earning men [7]. This counterintuitive result, also found in a previous Nordic studies [8], may reflect exposure misclassification for the cases or controls or self-selection of susceptible individuals into indoor professions or actual differences in sun protection behavior between outdoor workers and other workers.

Differences in sun protection behavior between outdoor and indoor workers at leisure and on sun holiday have not previously been studied as far as we know. We only know that individual use of sun protection varies considerably [9, 10] and that the solar UV radiation exposure of Danish outdoor and indoor workers differs significantly at work and is the same at leisure [11, 12] whereas sun holidays are more common at higher socioeconomic status in Danes and thus may be more frequent in indoor workers [13].

In Denmark, sun safety campaigns have failed to break the increasing curve of skin cancer incidence; skin cancer is the most common cancer and constitutes a rising health and socioeconomic problem [1, 14]. Sun safety campaigns that targets sun protection at work could work as an effective supplement to existing campaigns that mainly targets sun protection at leisure [15] and lead to better overall use of sun protection for the 400,000 Danish outdoor workers at risk. This would be analogous to the introduction of smoking ban at workplaces leading to an appropriate all-round modification of risk behavior in smoking—also a WHO group 1 carcinogen [16, 17].

Perception of disease risk typically motivates appropriate health behavior [18, 19]. This applies to skin cancer and use of sun protection at leisure and to some extend occupational skin cancer and use of sun protection at work [20–22]. However, a study by the Swedish Cancer Society showed that people had a tendency to underestimate skin cancer incidence in the population as well as the general health effects of skin cancer despite an otherwise realistic perception of skin cancer risk [23]. In addition, it is argued that perception of disease risk is only necessary to consider behavioral change and not sufficient to induce actual behavioral change [24].

The International Commission on Non-Ionizing Radiation Protection (ICNIRP) recommends workplace sun protection in form of long trousers and shirt with sleeves, a wide-brimmed hat, and sunscreen with protection factor 15–30 as well as shade seeking or breaks scheduled at solar noon [25].

Implementing the use of sun protection at work presents several challenges. Wearing long trousers, shirt with sleeves, and a wide brimmed hat while performing physical work in the summer causes increased body temperature leading to discomfort through heat sensation and physical exhaustion [26, 27]. Effective use of sunscreen requires a thick layer applied two or three times during working hours and is often perceived as a work hindrance due to stickiness [28, 29]. Shade seeking or breaks scheduled at solar noon impose financial and practical demands on employers [30].

The Danish occupational health and safety legislation have no requirements or operating guidelines on use of sun protection at work [31] and according to the health and safety organizations of major Danish contractors, general use of sun protection at Danish working sites is inadequate. This is somewhat in accordance with international studies on use of sun protection at work [32–42].

Most international studies on outdoor workers use of sun protection originate in Australia, where construction workers and lifeguard’s use of sun protection is decent but unrelated to their skin cancer knowledge [32–35]. A study from New Zealand reports coherence between workplace provision of sun-protective articles and actual use thereof [30]. Studies of outdoor workers in southern Europe find differences in attitudes about sun protection depending on profession. Attitudes were generally the best among farmers who nevertheless reported low use of sun protection, as did construction workers [36–42].

Females are better at overall use of sun protection, especially sunscreen [30, 41, 43–46]. Conversely, males wear a sunhat more frequently than females [43, 44, 46, 47]. Older age is associated with better overall use of sun protection [30, 48, 49]. Fairer skin type is associated with better overall use of sun protection and a greater risk of skin cancer [30, 33, 48, 50–52]. Construction and farm work are related to a greater risk of skin cancer [30, 33, 53, 54]. To our knowledge, there are no prior published Nordic behavioral studies on outdoor workers perception of occupational UV radiation exposure and skin cancer risk and use of sun protection.

We found only one scientific publication on behavioral differences at work and at leisure in outdoor workers; a Canadian study reporting significantly better use of shirt with sleeves and hat as well as slightly better overall sun protection behavior at work than at leisure [55]. We found no studies comparing outdoor and indoor workers use of sun protection at leisure and on sun holiday.

Several studies indicate that sun protection at work requires a sustained cooperative interdisciplinary effort; combining legislation, workplace policy, and provision of sun-protective articles as well as educational and role modeling actions [26, 30, 56, 57]. Australian employers may actually be held liable for photo damage as a result of UV radiation exposure due to inadequate sun protection at work [57].

The closer to the equator the more frequent the use of sun protection [14], which is appropriate in some respects. However, Northern country populations are generally more susceptible to UV radiation by virtue of having Caucasian skin types 1–3 on the Fitzpatrick scale with relatively little melanin and an increased risk of sunburn [58–60]. Therefore, sun protection is equally relevant in Northern European countries even if annual solar UV radiation is relatively lower compared to Southern European countries.

### Aim

The aim is to study the sun-protective behavior of Danish outdoor workers at work, at leisure, and on sun holiday and compare it to that of indoor workers.

## Methods

### Study design

This is a cross-sectional survey study of Danish outdoor and indoor workers’ skin cancer risk perception and sun protection behavior at work, at leisure, and on sun holidays.

### Setting

Recruitment by way of convenience sampling and data collection were carried out nationwide between April 2016 and April 2017. Danish workers were contacted by e-mail, posting on electronic media, in journals and at professional meetings in unions (3F, Dansk Byggeri, Asfaltindustrien), municipalities (Copenhagen, Middelfart, and Bornholm), company health and safety organizations (NCC, MT Højgaard, CG Jensen, Aarsleff, MALMOS, JORTON, Grøn Vækst, Copenhagen Malmö Port, Dansk Retursytem) as well as Holbæk and Roskilde Regional Hospitals and the Danish National Postal Service and volunteered as participants.

### Participant criteria

A seven-item electronic screening questionnaire and contact by telephone were used to screen the participants:

Inclusion criteria include occupational title as construction worker, roofer, paver, gardener, road worker, bricklayer, carpenter, unskilled laborer, farmer, sailor, postal worker or similar professions involving mainly outdoor or equal parts outdoor and indoor work or machinist, porter or similar professions with mainly indoor work.

Exclusion criteria include insufficient Danish language skills.

Written informed consent was obtained by all participants.

### Study population

The study population included 380 outdoor workers: mainly construction workers, gardeners, postal workers, roofers, and 119 indoor workers: mainly porters, carpenters and a variety of indoor professions including machinists. Based on the number of emails sent, readers of professional journals, and outdoor workers in recruitment companies and at meetings, the total number of study invitees during the recruitment process is estimated to be several thousands. We screened 531 workers who were interested in participating. Hereof two met the exclusion criteria and five did not met the inclusion criteria. Of the remaining 524 participants, nine withdrew their consent and 16 did not complete the study questionnaire for unknown reasons.

In addition, the participants were asked to wear an electronic UV-B dosimeter for personal measurements of solar UV radiation exposure at work and at leisure and offered a skin examination for clinical signs of skin cancer, actinic keratosis, and photoaging. The results thereof are reported elsewhere [13].

### Study questionnaire

The study questionnaire design was partly inspired by the questionnaire developed by Køster B. et al., as part of the Danish Sun Safety Campaign in 2013 [61]. However, most questions were new constructs developed for this particular study. The study questionnaire includes 47 items divided into distinct sections and are presented in the Appendix. Key term concepts such as summer season and around noon are defined in the questionnaire introduction as the period from May to September and the time between 11 AM and 3 PM.

Before use, the study questionnaire was reviewed by three experienced researchers and completed by six representative workers who were asked about wording and their understanding of specific items and key terms as well as overall layout and length of the study questionnaire, using a structured interview form. As consequence, questionnaire headlines were introduced making it easier to distinguish between work, leisure, and on sun holiday items on use of sun protection. The number of questionnaire items was reduced in order to shorten time spent on completion, and some questionnaire items were reworded into easier understandable terms. These changes resulted in an overall improvement in the questionnaire face validity. Completing the questionnaire required an e-mail address, internet access, and 10–15-min time. The Ramboll Survey XACT® module was used as survey tool.

### Definition of outdoor and indoor workers

Based on the question “What is your status as outdoor or indoor worker?”, participants were categorized as outdoor workers if they answered, “I predominantly work outdoor” or indoor workers if they answered, “I work equal parts outdoor and indoor” or “I predominantly work indoor”.

### Statistical analysis

Age was analyzed as mean. History of smoking, alcohol use, and sun holiday frequency answer categories were grouped in less detail for more clarity in that four different categories of daily tobacco use were grouped as one (current smoker), three different categories of weekly alcohol use above 10 units were grouped as one (more than 10 units/week) and six different categories of annual week spent on sun holiday were grouped into three (> 3 weeks, 1–3 weeks, and < 1 week). The actual categories used are presented in the Appendix.

Chi-square and Student’s *t* test were used to compare background characteristics for outdoor and indoor workers. Number and percentages were used to describe perception of occupational skin cancer risk and sun protection importance, availability of sun protection at work and challenges for its use, skin cancer risk factors and their relative importance, history of sunburn at work, and use of sun protection at work and at leisure/on sun holiday. Differences between use of sun protection at work and at leisure/on sun holiday in outdoor workers were tested using paired *t* tests and multiple regression models. Differences between outdoor and indoor workers’ use of sun protection at leisure and on sun holiday were tested using *t* tests and multiple regression models. Multiple regression model included adjustment terms for age, sex, educational level, history of smoking, and skin type. Effect measures are presented as Cohen’s *d*. Statistical significance was determined using *α* = .05, and all tests were two-sided. IBM SPSS version 24 (SPSS Inc., Chicago, IL, USA) was used for data analysis.

The Zealand Ethical Scientific Committee and Data Monitoring Authority approved the study (file numbers: SJ-509 and REG-130-2015).

## Results

Table 1 summarizes the characteristics for outdoor and indoor workers with statistical outcomes. Indoor workers had a significantly higher mean age, prevalence of higher education, and proportion of former smokers compared to outdoor workers who had a higher proportion of current smokers. For sex, history of skin or lip cancer, alcohol use, solarium use, skin type, weekly outdoor stay at leisure, and frequency of sun holidays, no significant differences were found between the two groups.

**Table 1:** Background characteristics for Danish outdoor and indoor worker

Covariates	Answers	Outdoor workers (*N* = 380)	Indoor workers (*N* = 119)	*p*
Mean age (SD), years	–	45.3	48.0	0.024^a^
Sex	Male	303 (79.7%)	92 (77.3%)	0.570
	Female	77 (20.3%)	27 (22.7%)	
Educational level	Elementary or vocational school	259 (68.2%)	66 (55.5%)	0.010
	Gymnasium	31 (8.2%)	10 (8.4%)	
	Higher education	65 (17.1%)	37 (31.1%)	
	Other	25 (6.6%)	6 (5.0%)	
History of skin or lip cancer	Yes	8 (2.1%)	4 (3.4%)	0.435
	No	372 (97.9%)	115 (96.6%)	
History of smoking	Never	202 (53.2%)	70 (58.8%)	0.006
	Former	79 (20.8%)	34 (28.6%)	
	Current	99 (26.0%)	15 (12.6%)	
Alcohol use	No	73 (19.2%)	17 (14.3%)	0.060
	Yes, less than 10 units/week	240 (63.2%)	89 (74.8%)	
	Yes, more than 10 units/week	67 (17.6%)	13 (10.9%)	
Solarium use, degree	None	159 (41.8%)	42 (35.3%)	0.433
	Low	142 (37.4%)	53 (44.5%)	
	Moderate	60 (15.8%)	20 (16.8%)	
	High	19 (5.0%)	4 (3.4%)	
Skin type	Type 1	10 (2.6%)	4 (3.4%)	0.858
	Type 2	102 (26.8%)	33 (27.7%)	
	Type 3	164 (43.2%)	53 (44.5%)	
	Type 4	86 (22.6%)	26 (21.8%)	
	Type 5	18 (4.7%)	3 (2.5%)	
Weekly outdoor stay at leisure	Less than 10 h	31 (8.2%)	7 (5.9%)	0.352
	Between 10 and 20 h	141 (37.1%)	55 (46.2%)	
	Between 20 and 30 h	123 (32.4%)	38 (31.9%)	
	Between 30 and 40 h	50 (13.2%)	12 (10.1%)	
	More than 40 h	35 (9.2%)	7 (5.9%)	
Sun holiday annual frequency	Less than 1 week	227 (59.7%)	57 (47.9%)	0.052
	Between 1 and 3 weeks	128 (33.7%)	49 (41.2%)	
	More than 3 weeks	25 (6.6%)	13 (10.9%)	

^a^Student’s *t* test, homogeneity of variance not assumed. Chi-squared test was used on all other covariates

Table 2 shows that among outdoor workers, near half (49.5%) do not think about the risk of occupational skin cancer, more than one in ten (11.8%) think the risk is insignificant or low and near one third (32.4%) think that use of sun protection at work is of low or no importance. Sun-protective measures made available at work are most commonly long trousers and shirt with sleeves (94.2%), followed by a wide-brimmed hat or cap (49.1%), sunscreen (29.6%), and avoiding the sun around noon (5.3%). In addition, employers attention to (22.1%) and information about (11.6%) use of sun protection at work is limited. The biggest challenges for using sun protection at work are that it is too hot (46.6%) or work disruptive (23.9%). However, very few outdoor workers (4.0%) consider the use sun protection at work as just not possible. Regarding beliefs around the risk factors for skin cancer, sunburn is considered important by 84.0% and the most important of those listed by 42.9% outdoor workers (not shown in table). Still, only about one in ten outdoor workers (11.1%) attest to never having been sunburned at work. More than eight in ten outdoor workers agree that the use of all types of sun protection reduces risk of skin cancer.

**Table 2:** Perception and attitude towards occupational skin cancer risk and sun protection

Covariates	Answers	Outdoor workers (*N* = 380)
Perception of occupational skin cancer risk	High	62 (16.3%)
	Moderate	85 (22.4%)
	Insignificant or low	45 (11.8%)
	Do not think about it	188 (49.5%)
Importance of sun protection at work	High	113 (29.7%)
	Moderate	144 (37.9%)
	Low or no	123 (32.4%)
Measures of sun protection made available at work *one or more*	Long trousers and shirt with sleeves	360 (94.2%)
	Wide-brimmed hat or cap	186 (49.1%)
	Sunscreen	112 (29.6%)
	Avoiding the sun around noon	20 (5.3%)
	General employer attention	84 (22.1%)
	Information	44 (11.6%)
Challenges for use of sun protection at work *agree or strongly agree one or more*	Too hot	177 (46.6%)
	Work disruptive	91 (23.9%)
	Spoils the pleasure of working outdoor	52 (13.8%)
	Too expensive	51 (13.4%)
	Just not possible	15 (4.0%)
	Risk of being ridiculed	9 (2.4%)
History of sunburn at work	Often	81 (21.3%)
	Rarely	257 (67.6%)
	Never	42 (11.1%)
General skin cancer risk factors considered important *one or more*	Sunburn	320 (84.2%)
	Solarium use	303 (79.7%)
	Sun holidays	222 (58.4%)
	Working outdoor	213 (56.1%)
	Outdoor stay at leisure	147 (38.7%)
Avoiding the sun around noon in the summer reduces risk of skin cancer	Agree	338 (88.9%)
	Disagree	15 (3.9%)
	Do not know	27 (7.1%)
Using sunscreen in the summer reduces risk of skin cancer	Agree	329 (86.6%)
	Disagree	15 (3.9%)
	Do not know	36 (9.5%)
Using a wide brimmed hat in the summer reduces risk of skin cancer	Agree	317 (83.4%)
	Disagree	15 (3.9%)
	Do not know	48 (12.6%)
Using long trousers and shirt with sleeves in the summer reduces risk of skin cancer	Agree	331 (87.1%)
	Disagree	16 (4.2%)
	Do not know	33 (8.7%)

Table 3 shows that outdoor workers avoid the sun around noon and use a wide brimmed hat or cap and sunscreen significantly more at leisure than at work. The opposite is true for use of long trousers and shirt with sleeves. For differences in outdoor workers use of sun protection at work and on sun holiday, the results are similar: avoid the sun around noon *d* = −.997, use a wide brimmed hat *d* = − .452, use of sunscreen *d* = − .853, and use long trousers and shirt with sleeves *d* = .693, *p* < 0.001. At leisure, indoor workers use sunscreen more often when compared with outdoor workers. On sun holiday, no differences in use of sun protection were found between outdoor and indoor workers. Results in multiple regression models adjusted for age, sex, educational level, history of smoking, and skin type reduced the effect size of difference estimates severely, caused by the number of included variables and the reduction in liable observations (reduction between 6% and 7%) leaving no significant comparisons between groups. Multiple regression model results are presented as Additional file 1: Table S1.

**Table 3:** Sun protective behavior at work, at leisure and on sun holidays

Covariates	Answers	At work / leisure				At leisure				On sun holiday			
		Outdoor workers (*N* = 380)	Outdoor workers (*N* = 380)	*p*	*d*	Outdoor workers (*N* = 380)	Indoor workers (*N* = 119)	*p*	*d*	Outdoor workers (*N* = 380)	Indoor workers (*N* = 119)	*p*	*d*
Avoid the sun around noon in the summer	Always	2 (0.5%)	3 (0.8%)	<.001	−.813	3 (0.8%)	5 (4.2%)	.168	.062	9 (3.0%)	3 (3.0%)	.288	.084
	Often	14 (3.7%)	115 (30.3%)			115 (30.3%)	36 (30.5%)			110 (37.0%)	47 (47.0%)		
	Rarely	143 (37.6%)	192 (50.5%)			192 (50.5%)	62 (52.5%)			141 (47.5%)	33 (33.0%)		
	Never	221 (58.2%)	70 (18.4%)			70 (18.4%)	15 (12.7%)			37 (12.5%)	17 (17.0%)		
Use a wide brimmed hat in the summer	Always	38 (10.0%)	30 (7.9%)	.002	−.162	30 (7.9%)	15 (12.8%)	.273	.049	30 (10.1%)	9 (9.1%)	1	0
	Often	58 (15.3%)	83 (21.8%)			83 (21.8%)	22 (18.8%)			92 (31.0%)	28 (28.3%)		
	Rarely	98 (25.8%)	129 (33.9%)			129 (33.9%)	46 (39.3%)			90 (30.3%)	42 (42.4%)		
	Never	186 (48.9%)	138 (36.3%)			138 (36.3%)	34 (29.1%)			85 (28.6%)	20 (20.2%)		
Use sunscreen in the summer	Always	33 (8.7%)	30 (7.9%)	.003	−.156	30 (7.9%)	19 (16.1%)	.032	.160	107 (35.9%)	42 (42.0%)	.402	.042
	Often	98 (25.8%)	113 (29.8%)			113 (29.8%)	38 (32.2%)			105 (35.2%)	33 (33.0%)		
	Rarely	163 (42.9%)	175 (46.2%)			175 (46.2%)	44 (37.3%)			54 (18.1%)	14 (14.0%)		
	Never	86 (22.6%)	61 (16.1%)			61 (16.1%)	17 (14.4%)			32 (10.7%)	11 (11.0%)		
Use long trousers and shirt with sleeves in the summer	Always	24 (6.3%)	6 (1.6%)	<.001	.565	6 (1.6%)	3 (2.5%)	.504	.023	9 (3.1%)	0 (0.0%)	−.231	.061
	Often	137 (36.1%)	72 (18.9%)			72 (18.9%)	25 (21.2%)			23 (7.8%)	15 (15.2%)		
	Rarely	180 (47.4%)	218 (57.4%)			218 (57.4%)	64 (54.2%)			145 (49.5%)	53 (53.5%)		
	Never	39 (10.3%)	84 (22.1%)			84 (22.1%)	26 (22.0%)			116 (39.6%)	31 (31.3%)		

*P*-values for comparisons in t-tests. Effect size given are Cohens *d*. Missing values are workers not spending outdoor leisure in Denmark or on sun holidays

Outdoor workers rarely or never use any type of sun protection at work (32.5%), at leisure (30.5%), and on sun holiday (13.9%). At leisure, outdoor workers use a wide brimmed hat or cap and long trousers and shirt with sleeves significantly more often compared to on sun holiday. The opposite is true for avoiding the sun around noon and use of sunscreen. The latter is by far the most commonly used form of sun protection on sun holiday. Sun protection of all types are used more often when made availability by employers at work (*p* < 0.001), except the use of long trousers and shirt with sleeves (*p* = 0.391). Long trousers and long-sleeved shirts are widely available (94.2%) but rarely or never used by outdoor workers at work (57.6%).

## Discussion

Our results show that Danish outdoor workers use sun protection significantly more at leisure and on sun holiday than they do at work and between outdoor and indoor workers, the only significant difference in UV exposure and use of sun protection outside working hours was for the use of sunscreen at leisure.

Many Danish outdoor workers do not consider the risk of occupational skin cancer or perceive the risk as low and rarely use sun protection at work. Under these circumstances, it is not surprising that most Danish outdoor workers have a history of sunburn at work.

Almost one third of outdoor workers rarely or never use any kind of sun protection. This applies to both at work and at leisure and reflects a sun protection behavior in line with their perception of occupational skin cancer risk and sun protection importance and contrary to the fact that most Danish outdoor workers agree that use of sun protection can reduce their risk of skin cancer.

Despite the challenges of using sun protection at work, only a minority of outdoor workers are actually opposed to it. In addition, judged by the willingness of several major Danish contractors to let their health and safety organizations contribute as active collaborators in this study, it seems that employers are positively prepared to offer their employees measures of sun protection at work.

Currently, only long trousers and shirt with sleeves are widely available from employers. However, in effect, less than half of the outdoor workers use long trousers and shirt with sleeves as sun protection at work, which is probably due to it being common workwear not intended as a sun protection measure and too hot to wear in the sun.

For comparison, a wide-brimmed hat or cap, sunscreen and avoiding the sun around noon are less often available from employers while availability and actual use are in fact significantly correlated for all of these at work.

The limited finding of behavioral differences in sun protection between outdoor and indoor workers in this study is unlikely to explain why Danish outdoor workers reportedly have low risk of skin cancer relative to the general population [7]. Furthermore, behavioral differences as regards UV radiation exposure at leisure, on sun holiday and by use of solarium between outdoor and indoor workers were not found in this study. Interestingly, sun holiday frequency was not positively associated with a significantly higher educational level in indoor workers in this study. Assuming that education level predicts socioeconomic status, this finding is contrary to results from previous Danish studies [11].

As is the case for outdoor workers in general, the use of sun protection is more frequent at leisure than at work for construction workers (*n* = 85) in this study. This is contrary to earlier findings in Canadian construction workers [53]. One possible explanations of this difference are more strict requirements for protective clothing and headgear safety use in Canada compared to Denmark [55].

People with high exposure to solar UV radiation often report themselves as having a higher skin type than that corresponding to their constitutive skin pigmentation [59]. In this study, outdoor workers show the same tendency to overestimate the sun protective properties of their own skin type, which further explains why almost nine out of ten have a history of sunburn at work. By comparison, in a 3-year prospective study, only half of Danish gardeners were sunburned [59] and in a population survey one third of Danish adults recalled at least one episode of sunburn in the last 12 months [10]. The marked difference in sunburn incidence is probably due to disparity in the sample period and population between the studies.

For preventive purposes, the more frequent use of sun protection at leisure and on sun holiday in Danish outdoor workers is something to build on as well as an indicator of the specific challenges of sun protection use at work. However, specially designed and commercially available UV protective clothing, hats, shading structures, and sunscreen can be applied to overcome some of the challenges [62–65].

Sunscreen is often the most used form of sun protection, albeit the least useful [10, 29, 57]. In this study, sunscreen was also the most used form of sun protection at leisure and especially so on sun holiday for outdoor and indoor workers alike. It is our opinion that the marketing of sunscreen and lack thereof for UV protective clothing and hats is the main reason for this. Aside from avoiding the sun around noon, the use of UV protective clothing and hat should be first priority as sun protection. Accordingly, the results of this study showing that avoiding the sun around noon at work and the use of long trousers and shirt with sleeves at leisure and on sun holiday is rare in Danish outdoor workers should be a matter of concern.

### Strengths and limitations

The strengths of the present study include a high diversity of professions in the study population, especially in the outdoor worker category, and thus a wider generalizability of study results.

Selection bias from worker self-selection or pre-screening may cause dispersal of results, making them unsuitable for generalization. In this study, the study population was sampled from a diverse selection of professions, broadly representing the intended target population.

Self-evaluated use of sun protection may lead to over- or underestimation.

Worksite observation of outdoor workers use of sun protection at work can be used as method to help validate our findings and describe the use of different measures of sun protection more in-depth.

By virtue of our choice of occupational titles, it is likely that higher educational level workers are poorly represented in the study population, especially so in indoor workers. Thus, somewhat careful consideration should be given to generalizing the results.

Of course, cross-sectional study findings are only present time, although in this context backwards representability seems plausible.

## Conclusions

This study strongly indicates that differences in the use of sun protection when exposed to UV radiation at leisure and on sun holiday between outdoor and indoor workers does not explain the, supposedly, lower risk of skin cancer in Danish outdoor workers relative to the general population, nor does there seem to be a decisive difference between Danish outdoor and indoor workers’ UV radiation exposure from weekly outdoor stay at leisure or sun holiday frequency. However, actual levels of UV radiation exposure should be measured objectively, using a technically and practically feasible method.

While risk of occupational skin cancer and the use of sun protection at work is largely neglected in Danish outdoor workers, they are still favorable towards the use of sun protection at work, especially so when provided by their employers. This indicates a great potential for introducing sun protection at work. The fact that outdoor workers protect themselves with greater care from the sun at leisure and on sun holiday and only differ from indoor workers by less use of sunscreen at leisure also suggests a singular prevention potential at work. Meanwhile, several major Danish contractors affirm that they are ready for taking part in responsibility for better sun protection at work by providing sun protection for the many thousands of Danish outdoor workers. However, currently, there is no legislation or guidelines for the use of sun protection at work to act on. Therefore, we propose the design of a commonsense operating guideline for the use of sun protection at work, as a joint effort between key stakeholders in the form of occupational medicine clinics, company health and safety organizations, worker unions, and The Danish Working Environment Authority.

### Additional file

Additional file 1: **Table S1.** Sun protective behavior at work, at leisure and on sun holidays; results of multiple regression. (DOCX 16 kb)

## Appendix

**Table:** Table 4 Study questionnaire items divided into sections

Sections	Questions and answer options (in italics)
Demographic characteristics 11 items	When were you born? - *1940, 1941, 1942…, 1999, 2000.* What is your sex? - *Male, female.* What is your smoking status? - *Nonsmoker, Former smoker, I smoke between 0 and 10 cigarettes a day, I smoke between 10 and 20 cigarettes a day, I smoke between 20 and 30 cigarettes a day, I smoke between 30 and 40 cigarettes a day, I smoke more than 40 cigarettes a day.* How much alcohol do you drink? - *I do not drink alcohol, between 0 and 10 items a week, between 10 and 20 items a week, between 20 and 30 items a week, more than 30 items a week.* Do you exercise regularly? - *Yes, no.* Where do you stay on holidays? - *I go on sun holiday more than 4 weeks a year, between 3 and 4 weeks a year, between 2 and 3 weeks a year, between 1 and 2 weeks a year, less than 1 week a year, I only spend my holidays in Denmark or neighboring countries* How many hours a week do you spend outdoor at leisure? - *More than 40 hours, between 30 and 40 hours, between 20 and 30 hours, between 10 and 20 hours, few hours, no hours* Have you used solarium? - *Yes to a very high extend, yes to a high extend, yes to moderate extend, yes to a low extend, yes to a very low extend, no never* How many hours a week do you spend outdoor at leisure? - *More than 40 hours, between 30 and 40 hours, between 20 and 30 hours, between 10 and 20 hours, few hours, no hours* Have you used solarium? - *Yes to a very high extend, yes to a high extend, yes to moderate extend, yes to a low extend, yes to a very low extend, no never* What is your skin type? - *According to the Fitzpatrick scale* [58]*.*
Occupational history 8 items	What is your highest educational level? - *Primary school, vocational school, gymnasium, higher education, other* What is your status as outdoor or indoor worker? - *I predominantly work outdoor, I work equal parts outdoor and indoor, I predominantly work indoor* Do your employer provide avoiding the sun around noon as sun protection at work? - *Yes, no* Do your employer provide long trousers and shirt with sleeves as sun protection at work? - *Yes, no* Do your employer provide a wide brimmed hat or cap (not safety helmet) as sun protection at work? - *Yes, no* Do your employer provide readily available sunscreen as sun protection at work? - *Yes, no* Do your employer provide information on prevention of sunlight exposure at work? - *Yes, no* Is your employer aware of protecting his employees from sunlight during outdoor work in the summer? - *Yes, no*
Attitude towards occupational skin cancer risk and use of sun protection 14 items	What do you think of the risk of occupational skin cancer? - *The risk is high, moderate, low, insignificant, I do not think about the risk* Do you think it is important to protect your skin from sunlight during outdoor work in the summer? - *Yes to a high degree, moderate degree, low degree, no* For me use of sun protection at work in the summer is: too hot, work disruptive, too expensive, spoils the pleasure of working outdoor, at risk of being ridiculed, just not possible? - *Strongly agree, agree, neither agree nor disagree, disagree, strongly disagree* Use of long trousers and shirt with sleeves in the summer can reduce my risk of skin cancer! - *Strongly agree, agree, disagree, strongly disagree.* Use of a wide brimmed hat in the summer can reduce risk of skin cancer! - *Strongly agree, agree, disagree, strongly disagree* Use of sunscreen in the summer can reduce risk of skin cancer! - *Strongly agree, agree, disagree or strongly disagree* Avoiding the sun around noon in the summer can reduce risk of skin cancer! - *Strongly agree, agree, disagree, strongly disagree* In your opinion which of the following are significant skin cancer risk factors? - *Sunburn, solarium use, sun holidays, working outdoor, outdoor stay at leisure* Of the following, which is the most significant skin cancer risk factor in your opinion? - *Sunburn, solarium use, sun holidays, working outdoor, outdoor stay at leisure*
Use of sun protection in the summer according to ICNRP guidelines 12 items	Do you use long trousers and shirt with sleeves respectively at work, at outdoor leisure in Denmark and on sun holiday to locations with more sun than in Denmark? - *Always, often, rarely, never, I do not spend outdoor leisure in Denmark, I do not go on sun holiday to locations with more sun than in Denmark.* Do you use a wide brimmed hat respectively at work, at outdoor leisure in Denmark and on sun holiday to locations with more sun than in Denmark? - *Always, often, rarely, never, I do not spend outdoor leisure in Denmark, I do not go on sun holiday to locations with more sun than in Denmark.* Do you use sunscreen respectively at work, at outdoor leisure in Denmark and on sun holiday to locations with more sun than in Denmark? - *Always, often, rarely, never, I do not spend outdoor leisure in Denmark, I do not go on sun holiday to locations with more sun than in Denmark.* Do you avoid the sun around noon respectively at work, at outdoor leisure in Denmark and on sun holiday to locations with more sun than in Denmark? - *Always, often, rarely, never, I do not spend outdoor leisure in Denmark, I do not go on sun holiday to locations with more sun than in Denmark.*
Health characteristics 2 items	Have you ever had skin or lip cancer? - *Skin cancer, lip cancer, skin and lip cancer, no, do not know.* How *often have you experienced a sunburn at work?* - *Often, rarely, never*

## Abbreviations

ICNRP: The International Commission on Non-Ionizing Radiation Protection
UV: Ultraviolet

## References

[1] Agner T, Ne E, Hc W, Jp B (2017). A scientific review addressing occupational skin cancer.

[2] John SM, Trakatelli M, Ulrich C (2016). Non-melanoma skin cancer by solar UV: the neglected occupational threat. J Eur Acad Dermatology Venereol.

[3] Lucas R, Van Deventer EP (2012). Solar ultraviolet radiation, assessing the environmental burden of disease at national and local levels.

[4] Bauer a, Diepgen TL, Schmitt J (2011). Is occupational solar ultraviolet irradiation a relevant risk factor for basal cell carcinoma? A systematic review and meta-analysis of the epidemiological literature. Br J Dermatol.

[5] Schmitt J, Seidler a, Diepgen TL, Bauer a (2011). Occupational ultraviolet light exposure increases the risk for the development of cutaneous squamous cell carcinoma: a systematic review and meta-analysis. Br J Dermatol.

[6] Milon A, Bulliard JL, Vuilleumier L, Danuser B, Vernez D (2014). Estimating the contribution of occupational solar ultraviolet exposure to skin cancer. Br J Dermatol.

[7] Kenborg L, Jørgensen AD, Budtz-Jørgensen E, Knudsen LE, Hansen J (2010). Occupational exposure to the sun and risk of skin and lip cancer among male wage earners in Denmark: a population-based case-control study. Cancer causes Control CCC.

[8] Håkansson N, Floderus B, Gustavsson P, Feychting M, Hallin N (2001). Occupational sunlight exposure and cancer incidence among Swedish construction workers. Epidemiology.

[9] Petersen B, Thieden E, Philipsen PA, Heydenreich J, Wulf HC, Young AR (2013). Determinants of personal ultraviolet-radiation exposure doses on a sun holiday. Br J Dermatol.

[10] Køster B, Thorgaard C, Philip A, Clemmensen IH (2010). Prevalence of sunburn and sun-related behaviour in the Danish population: a cross-sectional study. Scand J Public Health.

[11] Idorn LW, Wulf HC (2014). Socioeconomic status and cutaneous malignant melanoma in northern Europe. Br J Dermatol.

[12] Thieden E, Collins SM, Philipsen P a, Murphy GM, Wulf HC (2005). Ultraviolet exposure patterns of Irish and Danish gardeners during work and leisure. Br J Dermatol.

[13] Grandahl Kasper, Eriksen Paul, Ibler Kristina Sophie, Bonde Jens Peter, Mortensen Ole Steen (2018). Measurements of Solar Ultraviolet Radiation Exposure at Work and at Leisure in Danish Workers. Photochemistry and Photobiology.

[14] Dayan a D (1993). Solar and ultraviolet radiation. IARC monographs on the evaluation of carcinogenic risks to humans. Vol 55. J Clin Pathol.

[15] Reinau D, Weiss M, Meier CR, Diepgen TL, Surber C (2013). Outdoor workers’ sun-related knowledge, attitudes and protective behaviours: a systematic review of cross-sectional and interventional studies. Br J Dermatol.

[16] Fichtenberg CM, Glantz SA, Repace J, Lowrey A, Glantz S, Parmley W, Bero L (2002). Effect of smoke-free workplaces on smoking behaviour: systematic review. BMJ (Clinical Research Ed).

[17] Evans WN, Farrelly MC, Montgomery E (1999). Do workplace smoking bans reduce smoking?. Am Econ Rev.

[18] Brewer NT, Ph D, Weinstein ND, Cuite CL, Med AB (2004). Risk perceptions and their relation to risk behavior. Ann Behav Med.

[19] Conner M, Norman P (2005). Predicting health behaviour: Saf Sci.

[20] McMath BF, Prentice-Dunn S (2005). Protection motivation theory and skin cancer risk: the role of individual differences in responses to persuasive appeals. J Appl Soc Psychol.

[21] Janssen E, van Osch L, de Vries H, Lechner L (2011). Measuring risk perceptions of skin cancer: reliability and validity of different operationalizations. Br J Health Psychol.

[22] Dobbinson SJ, Wakefield MA, Jamsen KM (2008). Weekend sun protection and sunburn in Australia trends (1987–2002) and association with SunSmart television advertising. Am J Prev Med.

[23] Bränström R, Kristjansson S, Ullén H (2006). Risk perception, optimistic bias, and readiness to change sun related behaviour. Eur J Pub Health.

[24] Pligt J (1998). Perceived risk and vulnerability as predictors of precautionary behaviour. Br J Health Psychol.

[25] Protection ON, Workers OF, Radiation U (2010). ICNIRP statement--protection of workers against ultraviolet radiation. Health Phys.

[26] Nahar VK, Ford MA, Boyas JF, Brodell RT, Hutcheson A, Davis RE, Biviji-Sharma R (2014). Skin cancer preventative behaviors in state park workers: a pilot study. Environ Health Prev Med.

[27] Marlenga B (1995). The health beliefs and skin cancer prevention practices of Wisconsin dairy farmers. Oncol Nurs Forum.

[28] Laporte J (2006). Sensibilisation des salariés du bâtiment et des travaux publics au risque solaire: pour une prévention efficace. Arch Mal Prof Env.

[29] Shoveller JA, Lovato CY, Peters L, Rivers JK (2000). Canadian national survey on sun exposure & protective behaviours: outdoor workers. Can J Public Health.

[30] McCool JP, Reeder AI, Robinson EM (2009). Outdoor workers’ perceptions of the risks of excess sun-exposure. J Occup Health.

[31] The National Research Centre for the Working Environment Website. http://www.arbejdsmiljoviden.dk/nyt/nyheder/2017/april/04_pas-paa-foraarssolen. Accessed 15 July 2017.

[32] Gies P, Wright J (2003). Measured solar ultraviolet radiation exposures of outdoor workers in Queensland in the building and construction industry. Photochem Photobiol.

[33] Hammond V, Reeder AI, Gray AR (2008). Are workers or their workplaces the key to occupational sun protection?. Health Promot J Austr.

[34] Borland RM, Hocking B, Godkin GA (1991). The impact of a skin cancer control education package for outdoor workers. Med J Aust.

[35] Dobbinson S, Borland R, Anderson M (1999). Sponsorship and sun protection practices in lifesavers. Health Promot Int.

[36] Unverricht I, Knuschke P (2007). Verhalten von im Freien Beschäftigten gegenüber solarer UV-Strahlung in Beruf und Alltag. Dermatol Beruf Umwelt.

[37] Schmid-Kubista KE, Kellner L, Maier H (2010). Effect of work-related ultraviolet exposure and ophthalmic changes in Austrian farmers: the SVB-UV study. Ophthalmic Res.

[38] Maier H, Schmalwieser A, Rohn H (2009). UV-Belastung bei der ba¨uerlichen Arbeit.

[39] Milon A, Sottas PE, Bulliard JL (2007). Effective exposure to solar UV in building workers: influence of local and individual factors. J Expo Sci Environ Epidemiol.

[40] Madgwick P, Houdmont J, Randall R (2011). Sun safety measures among construction workers in Britain. Occup Med (Lond).

[41] Scerri L, Aquilina S, Amato GA (2002). Sun awareness and sun protection practices in Malta. J Eur Acad Dermatol Venereol.

[42] Zink A., Wurstbauer D., Rotter M., Wildner M., Biedermann T. (2017). Do outdoor workers know their risk of NMSC? Perceptions, beliefs and preventive behaviour among farmers, roofers and gardeners. Journal of the European Academy of Dermatology and Venereology.

[43] Lewis EC, Mayer JA, Slymen D (2006). Postal workers’ occupational and leisure-time sun safety behaviors (United States). Cancer Causes Control.

[44] Marrett LD, Pichora EC, Costa ML (2010). Work-time sun behaviours among Canadian outdoor workers: results from the 2006 National Sun Survey. Can J Public Health.

[45] Rosenman KD, Gardiner J, Swanson GM (1995). Use of skin-cancer prevention strategies among farmers and their spouses. Am J Prev Med.

[46] Schenker MB, Orenstein MR, Samuels SJ (2002). Use of protective equipment among California farmers. Am J Ind Med.

[47] Oliveira LMC, Glauss N, Palma A (2011). Hábitos relacionados à exposição solar dos professores de educação física que trabalham com atividades aquáticas. An Bras Dermatol.

[48] Duffy SA, Choi SH, Hollern R, Ronis DL (2012). Factors associated with risky sun exposure behaviors among operating engineers. Am J Ind Med.

[49] Popim RC, Corrente JE, Marino JAG, Souza CA (2008). Câncer de pele: uso de medidas preventivas e perfil demográfico de um grupo de risco na cidade de Botucatu. Ciênc Saúde Coletiva.

[50] Reeder AI, Gray A, McCool JP (2013). Occupational sun protection: workplace culture, equipment provision and outdoor workers’ characteristics. J Occup Health.

[51] Sena JS, Girão R, Carvalho S, Tavares RM, Fonseca FL, Silva P, Barbosa M (2016). Occupational skin cancer: systematic review. Revista Da Associação Médica Brasileira.

[52] Hall DM, McCarty F, Elliott T, Glanz K (2009). Lifeguards’ sun protection habits and sunburns: association with sun-safe environments and skin cancer prevention program participation. Arch Dermatol.

[53] Surdu S, Fitzgerald EF, Bloom MS, Boscoe FP, Carpenter DO, Haase RF (2013). Occupational exposure to ultraviolet radiation and risk of nonmelanoma skin cancer in a multinational European study. PLoS One.

[54] Simões TC, Souza NVDO, Shoji S, Peregrino AAF, Silva D (2011). Medidas de prevenção contra câncer de pele em trabalhadores da construção civil: contribuição da enfermagem. Rev Gaúcha Enferm.

[55] Peters CE, Koehoorn MW, Demers PA, Nicol AM, Kalia S (2016). Outdoor workers’ use of sun protection at work and leisure. Safety and Health at Work.

[56] Rye S, Janda M, Stoneham M, Crane P, Sendall M, Youl P, Thomas T, Louise B, Heather P, Linda F, Kimlin M (2014). Changes in outdoor workersʼ sun-related attitudes, beliefs, and behaviors. J Occup Environ Med.

[57] Horsham Caitlin, Auster Josephine, Sendall Marguerite C, Stoneham Melissa, Youl Philippa, Crane Phil, Tenkate Thomas, Janda Monika, Kimlin Michael (2014). Interventions to decrease skin cancer risk in outdoor workers: update to a 2007 systematic review. BMC Research Notes.

[58] Fitzpatrick TB (1975). Soleil et peau. J Med Esthet.

[59] Thieden E (2008). Sun exposure behaviour among subgroups of the Danish population. Based on personal electronic UVR dosimetry and corresponding exposure diaries. Dan Med Bull.

[60] Ravnbak MH (2010). Objective determination of Fitzpatrick skin type. Dan Med Bull.

[61] Køster B. Development and validation of scales and questionnaire for the monitoring and evaluation of Danes’ UV exposure as a scientific tool to reduce the prevalence of skin cancer: PhD-thesis, Research Unit of General Practice, Faculty of Health Sciences, University of Southern Denmark; 2016. p. 24–5. Retrieved from https://www.researchgate.net/profile/Brian_Koster2/publication/313064229_Development_and_validation_of_scales_and_questionnaire_for_the_monitoring_and_evaluation_of_Danes'_UV_exposure_as_a_13_978-87-93192-88-1/links/5a829e1645851504fb357293/Development-and-validation-of-scales-and-questionnaire-for-the-monitoring-and-evaluation-of-Danes-UV-exposure-as-a-scientific-tool-to-reduce-the-prevalence-of-skin-cancer-ISBN-13-978-87-93192-88-1.pdf. Accessed 19 Sept 2018.

[62] The Mascot UV protective work clothes Danish webshop. https://www.mascotwebshop.dk/uv-beskyttelse. Accessed 20 July 2017.

[63] The Smartsunwear UV protective headwear Danish webshop https://smartsunwear.dk/shop/6-solhatte/#!?page3. Accessed 19 Sept 2018.

[64] The Acabus shade structures Australian webshop. http://www.shadestructures.com.au/technical-data-installation.php#. Accessed 20 July 2017.

[65] The Cancercouncil International sunscreen webshop. https://www.cancercouncilshop.org.au/product/sunscreen-work-range.html. Accessed 20 July 2017.

