# Correction to: Skin cancer risk perception and sun protection behavior at work, at leisure, and on sun holidays: a survey for Danish outdoor and indoor workers

**DOI:** 10.1186/s12199-018-0749-5

**Published:** 2018-11-14

**Authors:** Kasper Grandahl, Kristina Sophie Ibler, Gunnar Hellmund Laier, Ole Steen Mortensen

**Affiliations:** 1 The Department of Occupational Medicine, Copenhagen University Holbaek, Gl. Ringstedvej 4B, 4300 Holbaek, Denmark; 2 The Department of Dermatology, Zealand University Hospital, Roskilde, Denmark; 3 PFI (Production, Research, Innovation) Region Zealand, Sorø, Denmark; 4 Section of Social Medicine, Department of Public Health, University of Copenhagen, Copenhagen, Denmark

## Correction to: Environmental Health and Preventive Medicine (2018) 23:47 DOI: 10.1186/s12199-018-0736-x

Following publication of the original article [1], it was highlighted that Table 3 contained an error. The *p*-value for use of long trousers and shirt with sleeves on sun holiday should not be preceded by a minus sign (−). The correct value is 231. This Correction article shows the correct Table 3. The original article has been updated.

**Table 3:** Sun protective behavior at work, at leisure and on sun holidays

Covariates	Answers	At work / leisure				At leisure				On sun holiday			
		Outdoor workers (*N* = 380)	Outdoor workers (*N* = 380)	*p*	*d*	Outdoor workers (N = 380)	Indoor workers (*N* = 119)	*p*	*d*	Outdoor workers (N = 380)	Indoor workers (N = 119)	*p*	*d*
Avoid the sun around noon in the summer	Always	2 (0.5%)	3 (0.8%)	<.001	−.813	3 (0.8%)	5 (4.2%)	.168	.062	9 (3.0%)	3 (3.0%)	.288	.084
	Often	14 (3.7%)	115 (30.3%)			115 (30.3%)	36 (30.5%)			110 (37.0%)	47 (47.0%)		
	Rarely	143 (37.6%)	192 (50.5%)			192 (50.5%)	62 (52.5%)			141 (47.5%)	33 (33.0%)		
	Never	221 (58.2%)	70 (18.4%)			70 (18.4%)	15 (12.7%)			37 (12.5%)	17 (17.0%)		
Use a wide brimmed hat in the summer	Always	38 (10.0%)	30 (7.9%)	.002	−.162	30 (7.9%)	15 (12.8%)	.273	.049	30 (10.1%)	9 (9.1%)	1	0
	Often	58 (15.3%)	83 (21.8%)			83 (21.8%)	22 (18.8%)			92 (31.0%)	28 (28.3%)		
	Rarely	98 (25.8%)	129 (33.9%)			129 (33.9%)	46 (39.3%)			90 (30.3%)	42 (42.4%)		
	Never	186 (48.9%)	138 (36.3%)			138 (36.3%)	34 (29.1%)			85 (28.6%)	20 (20.2%)		
Use sunscreen in the summer	Always	33 (8.7%)	30 (7.9%)	.003	−.156	30 (7.9%)	19 (16.1%)	.032	.160	107 (35.9%)	42 (42.0%)	.402	.042
	Often	98 (25.8%)	113 (29.8%)			113 (29.8%)	38 (32.2%)			105 (35.2%)	33 (33.0%)		
	Rarely	163 (42.9%)	175 (46.2%)			175 (46.2%)	44 (37.3%)			54 (18.1%)	14 (14.0%)		
	Never	86 (22.6%)	61 (16.1%)			61 (16.1%)	17 (14.4%)			32 (10.7%)	11 (11.0%)		
Use long trousers and shirt with sleeves in the summer	Always	24 (6.3%)	6 (1.6%)	<.001	565	6 (1.6%)	3 (2.5%)	504	.023	9 (3.1%)	0 (0.0%)	.231	.061
	Often	137 (36.1%)	72 (18.9%)			72 (18.9%)	25 (21.2%)			23 (7.8%)	15 (15.2%)		
	Rarely	180 (47.4%)	218 (57.4%)			218 (57.4%)	64 (54.2%)			145 (49.5%)	53 (53.5%)		
	Never	39 (10.3%)	84 (22.1%)			84 (22.1%)	26 (22.0%)			116 (39.6%)	31 (31.3%)		

*P*-values for comparisons in t-tests. Effect size given are Cohens *d.* Missing values are workers not spending outdoor leisure in Denmark or on sun holidays
