# Supplementary material for: Skin cancer risk perception and sun protection behavior at work, at leisure, and on sun holidays: a survey for Danish outdoor and indoor workers
Source: Environ Health Prev Med. 2018 Oct 2;23:47. doi: 10.1186/s12199-018-0736-x (PMC6169023; doi:10.1186/s12199-018-0736-x)
Supplement: Supplementary file 1 — Table S1. Sun protective behavior at work, at leisure and on sun holidays; results of multiple regression. (DOCX 16 kb) [file 12199_2018_736_MOESM1_ESM.docx]

| **Covariates** | **Settings** | N | df | t | *d* | p |
| --- | --- | --- | --- | --- | --- | --- |
| **Avoid the sun around noon in the summer** | At work vs. leisure  At work vs. sun holiday  At leisure  On sun holiday | 355 | 344 | 1.162 | .063 | .246 |
|  |  | 278 | 268 | .743 | .045 | .458 |
|  |  | 467 | 455 | .237 | .011 | .813 |
|  |  | 373 | 361 | -.326 | -.017 | .744 |
| **Use a wide brimmed hat in the summer** | At work vs. leisure  At work vs. sun holiday  At leisure  On sun holiday | 355 | 344 | .250 | .013 | .803 |
|  |  | 278 | 268 | .196 | .012 | .845 |
|  |  | 466 | 454 | .359 | .017 | .720 |
|  |  | 372 | 360 | -.850 | -.045 | .396 |
| **Use sunscreen in the summer** | At work vs. leisure  At work vs. sun holiday  At leisure  On sun holiday | 354 | 343 | .977 | .053 | .329 |
|  |  | 279 | 269 | -1.154 | -.070 | .249 |
|  |  | 466 | 454 | 1.766 | .083 | .078 |
|  |  | 374 | 362 | .468 | .025 | .640 |
| **Use long trousers and shirt with sleeves in the summer** | At work vs. leisure  At work vs. sun holiday  At leisure  On sun holiday | 355 | 344 | .247 | .013 | .805 |
|  |  | 274 | 264 | 1.740 | .072 | .083 |
|  |  | 467 | 455 | .001 | <.001 | .999 |
|  |  | 368 | 356 | .232 | .012 | .817 |

**Table S1** Sun protective behavior at work, at leisure and on sun holidays; results of multiple regression.

Control variables in the model are age, sex, educational level, history of smoking and skin type.
